# Divergent driving mechanisms of community temporal stability in China's drylands

**DOI:** 10.1016/j.ese.2024.100404

**Published:** 2024-03-01

**Authors:** Kai Wang, Cong Wang, Bojie Fu, Jianbei Huang, Fangli Wei, Xuejing Leng, Xiaoming Feng, Zongshan Li, Wei Jiang

**Affiliations:** a State Key Laboratory of Urban and Regional Ecology, Research Center for Eco-Environmental Sciences, Chinese Academy of Sciences, Beijing, 100085, China; b College of Resources and Environment, University of Chinese Academy of Sciences, Beijing, 100049, China; c Max Planck Institute for Biogeochemistry, Hans-Knöll-Str. 10, 07745, Jena, Germany; d Key Laboratory of Ecosystem Network Observation and Modeling, Institute of Geographic Sciences and Natural Resources Research, Chinese Academy of Sciences, Beijing, 100101, China; e Shaanxi Yan'an Forest Ecosystem National Observation and Research Station, Beijing, 100085, China; f National Observation and Research Station of Earth Critical Zone on the Loess Plateau in Shaanxi, Xi'an, 710061, China

**Keywords:** Dryland ecosystems, Community stability, Ecosystem management, Species richness, Aridity threshold

## Abstract

Climate change and anthropogenic activities are reshaping dryland ecosystems globally at an unprecedented pace, jeopardizing their stability. The stability of these ecosystems is crucial for maintaining ecological balance and supporting local communities. Yet, the mechanisms governing their stability are poorly understood, largely due to the scarcity of comprehensive field data. Here we show the patterns of community temporal stability and its determinants across an aridity spectrum by integrating a transect survey across China's drylands with remote sensing. Our results revealed a U-shaped relationship between community temporal stability and aridity, with a pivotal shift occurring around an aridity level of 0.88. In less arid areas (aridity level below 0.88), enhanced precipitation and biodiversity were associated with increased community productivity and stability. Conversely, in more arid zones (aridity level above 0.88), elevated soil organic carbon and biodiversity were linked to greater fluctuations in community productivity and reduced stability. Our study identifies a critical aridity threshold that precipitates significant changes in community stability in China's drylands, underscoring the importance of distinct mechanisms driving ecosystem stability in varying aridity contexts. These insights are pivotal for developing informed ecosystem management and policy strategies tailored to the unique challenges of dryland conservation.

## Highlights

• The driving mechanisms of community stability shifted at an aridity level of ∼0.88.
• Species richness has opposite roles in stability above and below the aridity threshold.
• Soil properties are important for the maintenance of stability.
• Different policies need to be implemented above and below the aridity threshold.

## Graphical abstract

**Figure:**
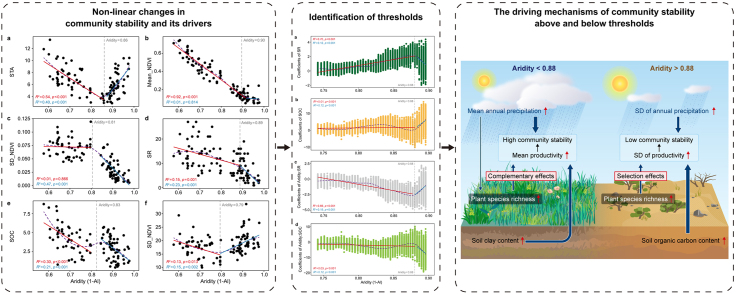

## 1 Introduction

Drylands, defined as regions with an aridity index (AI, i.e., the ratio of average annual precipitation to average annual potential evapotranspiration) below 0.65, occupy 41% of the Earth's land area and support more than 38% of the world human population [1]. Most drylands are characterized by low mean precipitation and high precipitation variability, relatively poor soils, sparse vegetation, and fragile ecosystems [2]. It is predicted to experience more extreme climate events and intense aridity [3,4]. The intensification of aridity and the reduction in rainfall in dryland ecosystems have changed the life form of dominant plant species (e.g., from herbs to shrubs) [5], reduced vegetation coverage [6] and soil microbial diversity [7], and decoupled the soil nutrient cycle [6], which may lead to a decline in ecosystem function.

The adverse effects of aridity intensification on dryland ecosystems depend on the ability of the ecosystem to adapt to environmental changes, that is, ecosystem stability. Ecosystem stability refers to the ability of an ecosystem to maintain or be restored to its original state after being disturbed and is one of the basic attributes of an ecosystem [8]. A study has shown that increasing aridity may even lead to systematic and abrupt changes in plant productivity, soil fertility, and plant cover and richness when aridity levels (1-AI, i.e., higher values indicated drier conditions) more than 0.54, 0.7, and 0.8, respectively [9]. Considering the ecosystem attributes may undergo nonlinear and abrupt changes along the aridity gradient, it is significant to examine dryland ecosystems' stability response to increasing drought.

The community's temporal stability depends on biodiversity, climate, and soil conditions [10,11]. Biodiversity mainly enhances community temporal stability through three main mechanisms: portfolio effects, overyielding effects, and species asynchrony [[12], [13], [14]]. Portfolio effects suggest more species increase community stability due to diverse population dynamics, minimizing the impact of individual population fluctuations [14]. Overyielding effects indicate that higher diversity boosts productivity, mitigating the impact of statistical stochasticity on the entire community and enhancing stability [12]. The species asynchrony theory proposes that community species exhibiting diverse responses to environmental disturbances due to different attributes [15] create temporal niche partition, consequently reducing overall community fluctuation [13].

Furthermore, biodiversity enhances productivity mainly through complementary effects (i.e., increased species diversity improves overall resource utilization efficiency) and selection effects (i.e., interaction-induced dominance of high-productivity species), thereby affecting the stability of community productivity through portfolio effects or overyielding effects [14,[16], [17], [18]]. As ecological niche complementation promotes species coexistence, it will produce stronger portfolio effects, overyielding effects, and community stability [14,19]. In contrast, selection effects tend to increase the dominance of high-yielding species, thereby reducing species evenness and decreasing portfolio effects and community stability [14,19]. Through the community model, Wang et al. [18] also indicated that complementary effects enhance stability by increasing portfolio effects, while selection effects diminish stability by selecting species with high productivity but low tolerance.

Similarly, abiotic factors such as climate and soil conditions also significantly impact community stability. Climate change can alter ecosystem functions, cause biodiversity loss and species composition changes, increase ecosystem vulnerability, and threaten ecosystem production [20]. In fact, recent studies have found that climate warming and the decrease in annual precipitation may lead to a decrease in the temporal stability of plant community biomass production by altering the species dynamics of the plant community [21,22]. Moreover, extreme climate events, such as daily temperature and precipitation extremes, have changed in intensity and frequency over recent decades [23]. The increase in climate variability, for example, precipitation variability, may also reduce community stability [20]. In addition, local soil conditions also impact community stability by affecting ecological factors. Previous studies suggest that local soil conditions, especially soil organic matter, can affect community stability directly by increasing mean net primary productivity more quickly than its temporal variability, and the direct impact of climate on stability is lower than that of local soil conditions [11]. Furthermore, interactions of climate change, soil conditions, and biodiversity make the maintenance mechanism of community stability more complex [10,11].

Ecosystem attributes are highly correlated, and changes in a given attribute caused by climate change may trigger changes in other attributes that rely on this attribute but operate at different spatiotemporal scales [2]. The response of ecosystem attributes to climate change may exist thresholds, and the climate threshold that causes a sudden change in a certain attribute may trigger changes in a range of related ecological attributes [9,24]. Specifically, the response of community stability to climate change may undergo sudden changes beyond a certain threshold, which may also cause changes in plant and soil attributes that affect community stability. Therefore, identifying this threshold helps us understand these chain changes and clarify the driving mechanisms of community stability more clearly. Recent studies on mechanisms driving ecosystem stability have mainly come from local-scale experiments, where the included species have been randomly selected, and stability has been assessed under limited environmental conditions [25]. Especially in fragile dryland ecosystems, research on the interactive mechanisms that underlie ecosystem stability is limited.

China has approximately 6.6 million km^2^ drylands, with significant environmental and vegetation differences [26,27]. These drylands are vulnerable and sensitive to environmental change [28]; thus, several land protection and ecological restoration projects have been implemented to mitigate land degradation in China's drylands since the 1970s [29]. However, these large-scale projects increased the pressure on water supplies, thus exacerbating the tradeoff between carbon and water [30,31]. Ecosystem stability enables the ecosystem to maintain relative resilience in the face of pressures such as natural disasters, human disturbances, or climate change [32], which would play a crucial buffering role in addressing ecosystem abrupt and possibly irreversible shift in large-scale ecological projects. Due to increased aridity, there is also a risk of dryland expansion. The stability of the ecosystem is a determining factor in whether degradation of dryland ecosystems will occur and whether vegetation restoration efforts will be sustainable.

Ecosystem stability is a multifaceted and multidimensional concept with diverse metrics in practical studies [33], while it is usually measured in terms of the temporal stability of ecosystem functions. This term is commonly associated with either the ability of an ecosystem to reduce the variability of one of its components over time or recover it quickly after a disturbance, primarily characterized by temporal variability, resistance, and recovery [32]. The quantification of an ecosystem stability typically usually involves using the temporal variability index in a particular ecosystem function over time, as well as the resistance index and resilience index when facing external pressures [34]. Most studies have focused on the temporal stability of community productivity, defined by the ratio of time-mean biomass to its standard deviation [35]. Thus, we used NDVI as the proxy of community productivity and defined community stability as the ratio of the mean annual peak NDVI to its standard deviation [10]. The main objective is to analyze the variation of community stability along an aridity gradient in the drylands of China and explore whether there was an aridity threshold leading to a nonlinear variation in community stability. Combining the transect survey data with satellite data, we further analyzed the underlying driving mechanisms of species richness, climate, and soil properties on community stability.

## 2 Materials and methods

### 2.1 Study area

The study area is located in the dryland ecosystems of northern China (Fig. S1), with a latitudinal range from 31°42′ to 53°23′ N and a longitudinal range from 73°40′ to 126°04′ E. The whole region is located inside the Eurasian continent, with a dry climate, large annual temperature ranges, and windy weather. The annual precipitation ranges from 21 to 453 mm, and the average annual temperature ranges from −4 to 13 °C. The vegetation types from east to west are meadow grassland (dominated by *Stipa* spp. and *Leymus* spp.), typical grassland (dominated by *Stipa* spp., *Leymus* spp., and *Cleistogenes* spp.), scrub (dominated by *Stipa* spp. and *Nitraria* spp.), desert grassland (*Stipa* spp., *Reaumuria* spp., *Calligonum* spp., and *Nitraria* spp.) and desert (*Reaumuria* spp., *Calligonum* spp., *Nitraria* spp., and *Haloxylon* spp.). The survey sites in the study area encompass the 14 soil types, i.e., Aeolian soil, Alluvial soils, Brown desert soil, Brown pedocals, Castanozems, Cultivated loessial soils, Desert solonchaks, Fluvo-aquic soils, Gray desert soils, Gray-brown desert soils, Litho soils, Meadow soils, Sierozems, and Skeletol soils (https://www.resdc.cn/).

### 2.2 Field community survey

Along the aridity gradient, we selected sampling sites suitable for community surveys. At each sampling site, we set one 45 × 45 m sample plot conducting plant diversity surveys and collecting soil samples during the peak growing season (July–August) in 2020 and 2021, either grassland or shrubland, depending on the dominant ecosystem in each survey site, and the latitude and longitude of the plot were recorded. We set 45 sample points along the aridity gradient. These plots were selected as far away as possible from the impact of human activities and other disturbances on plants and soil. Four 30-m-long sample lines with 10-m intervals were laid in each sample plot, and five 1 × 1 m survey quadrats were randomly set on each sample line. All species occurring in the quadrats were surveyed and recorded, and plant species richness was quantified as the sum of species in all quadrats [1]. To understand the survival strategies of plants, we measured the specific leaf area (SLA) of the dominant species in each sample plot. The SLA is the ratio of leaf area to leaf dry weight. At each sampling plot, we randomly selected 3–5 individuals for each plant species, and from each individual, we randomly sampled 3–5 leaves. The leaf area of each dominant species was measured with a leaf area meter (Yaxin-1241), and the leaf dry weight was measured after drying at 75 °C to constant weight. The community-weighted SLA (weighted by relative cover) represents the SLA of the entire community. Soil cores with a diameter of 6 cm were collected at a depth of 0–30 cm on the upper, middle, and lower slopes of each sample plot and brought to the laboratory to measure soil properties.

To expand the sample size, the species richness and soil properties from four related studies were collected using ISI Web of Science and China National Knowledge Infrastructure (Supporting Information Data S1; a list of the data sources is given in Supporting Information Appendix S1). The ecosystems involved in these four studies are all-natural grassland or shrubland ecosystems, and the quadrat size is the same as ours. We obtained the table-form data directly and extracted graphical data using Get Data Graph Digitizer 2.20 [36]. The total number of sampling plots was 109, including 48 sample points in the semiarid region, 53 in the arid region, and 8 in the hyper-arid region.

### 2.3 Climatic and soil variables

Meteorological data were obtained from the China Meteorological Data Network (http://data.cma.cn/), and multiyear temperature and multiyear precipitation were obtained by spatial interpolation of relevant meteorological data from 2000 to 2016 at meteorological stations near the sampling site. Potential evapotranspiration was calculated for each site by the Penman-Monteith formula [37]. Based on the above variables, we calculated the aridity index (AI = precipitation/potential evapotranspiration), widely used to measure the degree of aridity worldwide [25]. To facilitate the interpretation of the results, we used 1-AI that expressed in terms of aridity level to represent the level of aridity in our analysis, i.e., higher values indicated drier conditions. To assess climate change, the following four indicators were used: (ⅰ) mean annual precipitation, (ⅱ) interannual precipitation variability (standard deviation of annual precipitation), (ⅲ) mean annual temperature, and (ⅳ) interannual temperature variability (standard deviation of annual temperature), which were the main climate drivers used to assess terrestrial net primary productivity [38]. We used soil organic carbon and soil clay to assess the soil properties at each site, as these soil properties play a key role in water availability and plant growth and are important drivers of plant diversity and ecosystem function in dryland ecosystems [39]; additionally, they tended to be relatively constant over the time scales considered in the paper [40]. The soil organic carbon was determined using the potassium dichromate volumetric method [1], and the soil clay was extracted from the SoilGrids system (https://soilgrids.org/).

### 2.4 Community productivity stability

Since obtaining continuous community productivity data for a long time series is difficult, remote sensing provides a feasible way to solve this problem [10]. We used the NDVI to represent the aboveground net primary productivity of the community. The NDVI characterizes vegetation cover, is linearly correlated with photosynthetically active radiation, and is considered a good proxy for aboveground biomass [41]. The NDVI data for each site were acquired using the MOD13Q1 product from the Moderate Resolution Imaging Spectroradiometer (https://daac.ornl.gov/), which provides data 23 times per year (every 16 days) at a pixel size of 250 × 250 m. We used the platform Google Earth Engine and extracted the maximum value of NDVI from the Sentinel-2 Multispectral Instrument (pixel size of 10 × 10 m) from 1 January 2016 to 31 December 2016. We calculated the average value of NDVI from a 3 × 3 matrix of pixels (similar to the size of the community survey) centered on each site location and compared it with the NDVI (250 × 250 m) from MOD13Q1. We detected a close relationship between both pixel sizes (Fig. S2), indicating that the areas of community survey were sufficiently homogeneous to avoid scale mismatch between field and remote sensing data. The peak NDVI within each year from 2000 to 2016 was used as a proxy for community productivity in that year, and the temporal stability of community productivity was calculated as follows:

(1) $$\text{Stability}=\frac{\mu }{\delta }$$

where *μ* and *δ* are the annual mean peak NDVI calculated from 2000 to 2016 and the standard deviation (SD) of the annual peak NDVI over that period, respectively.

### 2.5 Statistical analyses

We selected species richness, annual mean and interannual variability of temperature and precipitation, soil organic carbon, and soil clay content to characterize the biodiversity, climate, and soil properties, respectively, and further explored the effects of these environmental and ecological factors on community stability.

#### 2.5.1 Evaluation of non-linear responses to aridity

We used the locally weighted regression to fit the nonlinear changes of community stability and ecological factors along the aridity gradient. We used the segmented linear regression model to identify tipping points by the “segmented” package [42]. When there are multiple response states of the corresponding variable with the change of the independent variable, they are difficult to explain by one regression model. Segmented regression can find the appropriate breakpoint location according to the response state, thus dividing the independent variable into a limited number of intervals and describing the relationship between them separately in different intervals [42].

#### 2.5.2 Thresholds detection

Considering that the variables exhibited non-linear changes along the aridity gradient, we established a linear mixed-effects model to test the relationships between the species richness or soil organic carbon and the community stability using “lme4” and “lmerTest” packages [43,44]:

Communitystability∼Aridity+Speciesrichness+Soilorganiccarbon+SoilClaycontent+Aridity×Speciesrichness+Aridity×Soilorganiccarbon+Aridity×Speciesrichness×Soilorganiccarbon+(1|Soil_type)+(1|Vegetation_type),

where “ × ” indicates an interaction term.

To evaluate how each explanatory factor affected stability along the aridity gradient, we conducted a moving-window analysis for the linear mixed-effects model using “parallel” and “doSNOW” packages [45]. Specifically, we first ordered all the sites surveyed according to aridity. Then, we took the 65 sites (this number of sites provided sufficient statistical power for our model) with the lowest values of aridity and performed the linear mixed-effects model. We then extracted the effect value of species richness or soil organic carbon on community stability. To improve the robustness of the results, we applied the bootstrap method to bootstrap the standardized slopes of each predictor to obtain their confidence intervals, which were matched to the average value of aridity across 65 selected sites. Next, we removed the community with the lowest value of aridity from the selected sites and added the community scoring the next higher value to repeat the same calculations. We repeated this loop as many times as sites remained (i.e., 44). Based on the above-extracted effect values, we constructed the relationship between the effect value and aridity and used segmented linear regression to identify the aridity threshold.

#### 2.5.3 Identification of relationships among various factors

To identify the direct and indirect effects of these ecological factors on community stability above and below the threshold, we used the piecewise structural equation modeling based on the directional separation method. Structural equation modeling (SEM) was a probability model that integrated multiple prediction factors and response variables in a causal network. Compared with the standard SEM, the piecewise structural equation modeling allowed us to relax some limitations, including nonnormal data, nonlinear relationships between variables, and small sample sizes [46]. We used direct separation tests based on Fisher's C statistic to assess overall model fit, with the model being accepted when *p* > 0.05. Fisher's C and *p*-values are both statistical measures used to assess the overall fit of a model to the data [46]. Generally, when *p* is greater than 0.05, the model can be accepted, and a higher Fisher's C indicates a better overall model fit. The piecewise structural equation models were constructed using the "PiecewiseSEM" package [46]. Before performing SEM operations, the variables were log-transformed or square root-transformed to meet homogeneity and normality requirements.

All the above analyses were performed using R 4.1.1 [47].

## 3 Results

### 3.1 The responses of community stability and environmental and ecological factors to aridity

**Fig. 1:**
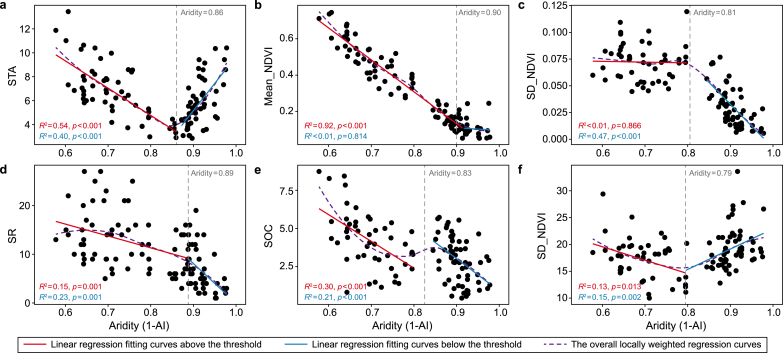
The response of community stability and its components, species richness, and soil properties with increasing aridity. Nonlinear variation in STA (community temporal stability, **a**), Mean_NDVI (the mean peak NDVI, **b**), SD_NDVI (the SD of the peak NDVI, **c**), SR (species richness, **d**), SOC (soil organic carbon, **e**), and clay (soil clay, **f**) with increasing aridity and their aridity thresholds. The red and blue solid lines indicate the linear regression fitting curves above and below the threshold, respectively, and the purple dashed line indicates the overall locally weighted regression curves.

The changes in community stability along the aridity gradient showed a U-shaped curve, decreasing and then increasing with increasing aridity; shifts occurred at an aridity level of 0.86 (Fig. 1a). The annual mean peak NDVI and the SD of the peak NDVI, which are components of community stability, turned at an aridity level of 0.90 and 0.81, respectively, where the mean peak NDVI decreased with increasing the aridity level above 0.90 and did not change with the aridity level below 0.90 (Fig. 1b). The SD of the peak NDVI did not vary with aridity until an aridity level of 0.81 and decreased with increasing the aridity level below 0.81 (Fig. 1c). Species richness and soil organic carbon content turned at an aridity level of 0.89 and 0.83, respectively. Their reduction rates were faster below the turning point than above (Fig. 1d and e). Soil clay content showed a U-shaped curve, turning at an aridity level of 0.79 (Fig. 1f). Moreover, the mean annual precipitation and the SD of the annual precipitation decreased significantly with increasing aridity (Fig. S3a and b). The mean annual temperature also showed a U-shaped curve on the aridity gradient, turning at an aridity level of 0.72 (Fig. S3c), while the SD of annual temperature did not change with aridity (Fig. S3d).

### 3.2 The aridity threshold causing a sudden change in community stability

**Fig. 2:**
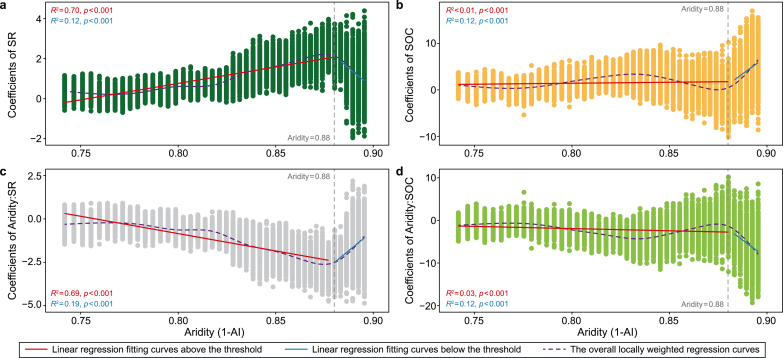
The effects of species richness, soil organic carbon, interaction between species richness and aridity, and interaction between soil organic carbon and aridity on community stability. Nonlinear changes of coefficients of species richness (**a**), soil organic carbon (**b**), interaction between species richness and aridity (**c**), and interaction between soil organic carbon and aridity (**d**) on community stability obtained from a linear-effects model throughout a moving subset window. SOC, soil organic carbon; SR, species richness; “:”, interaction. The red and blue solid lines indicate the linear regression fitting curves above and below the threshold, respectively, and the purple dashed line indicates the overall locally weighted regression curves.

The effects of species richness, soil organic carbon, interaction between species richness and aridity, and interaction between soil organic carbon and aridity on community stability all showed abrupt changes when the aridity level was 0.88 (Fig. 2a–d). Thus, it is no surprise that the aridity threshold for sudden change in driving mechanisms of community stability was 0.88. Specifically, the effect value of species richness on community stability increased when the aridity level was less than 0.88 and then decreased with aridity. In contrast, the effect value of interaction between species richness and aridity on community stability decreased when the aridity level was less than 0.88 and then increased with aridity. Moreover, the effect value of soil organic carbon on community stability increased with aridity. Yet, the slope of the relationship between soil organic carbon and community stability was larger when the aridity level was more than 0.88. However, the effect value of interaction between soil organic carbon and aridity on the community along the aridity gradient was opposite to that of soil organic carbon.

### 3.3 Direct and indirect effects of climate, species richness, and soil properties on community stability

**Fig. 3:**
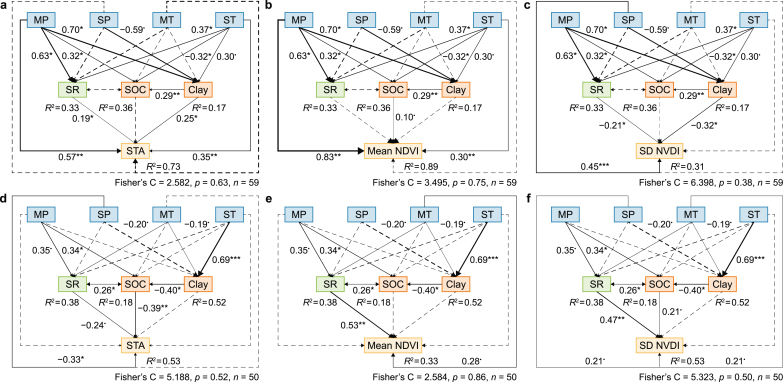
Relationships between climate, species richness, soil properties, and community stability and its components. **a**–**c**, Aridity <0.88, direct and indirect effects of climate, species richness, and soil properties on community stability (STA, **a**), the mean peak NDVI (Mean_NDVI, **b**), and the SD of the peak NDVI (SD_NDVI, **c**). **d**–**f**, Aridity>0.88, direct and indirect effects of climate, species richness, and soil properties on community stability (STA, **d**), the mean peak NDVI (Mean_NDVI, **e**), and the SD of the peak NDVI (SD_NDVI, **f**). The black continuous arrows and black dashed arrows indicate statistically significant and insignificant path coefficients between variables, respectively, and the width of the arrows indicates the strength of the relationship between variables, as measured by the standardized path coefficients. ∗∗∗*p* < 0.001, ∗∗*p* < 0.05, ∗*p* < 0.01, ˙*p* < 0.1.

In regions with aridity levels <0.88, SEM accounted for 72%, 87%, and 28% of the variation in community stability, mean peak NDVI and SD of peak NDVI, respectively (Fig. 3a–c). The effects of explanatory factors on community stability mainly acted on the mean peak NDVI. Specifically, plant species richness had a positive effect on community stability by reducing the SD of the peak NDVI (Fig. 3c). High precipitation and soil clay content contributed to greater stability, and high precipitation indirectly enhanced community stability through increased species richness (Fig. 3a). Soil organic carbon had a weak effect on both mean and SD of peak NDVI, insignificantly affecting community stability (Fig. 3b–c, *p* > 0.1).

In regions with aridity levels >0.88, SEM explained 53%, 39%, and 53% of the variation in community stability, mean peak NDVI, and SD of peak NDVI, respectively (Fig. 3d–f). The effects of explanatory factors on community stability mainly acted on the SD of the peak NDVI. The SD of precipitation directly influenced the SD of peak NDVI, indicating that communities with a small SD of precipitation were more stable (Fig. 3f). While species richness negatively impacted the SD of peak NDVI, its overall effect on community stability was not significant (Fig. 3d, *p* > 0.1). Plant communities with high mean annual precipitation and low SD of temperature had high soil organic carbon, which, in turn, reduced community stability by increasing the SD of peak NDVI (Fig. 3f). Soil clay indirectly affected community stability through its impact on soil organic carbon (Fig. 3d).

## 4 Discussion

### 4.1 Impacts of climate change, biodiversity, and soil properties on community stability

Some studies have explored the impact of ecological factors such as climate change and biodiversity on community stability [20,22] and have also discovered different driving mechanisms of community stability under climate thresholds [25]. For example, a study on the global dryland ecosystems stability shows that under a low aridity level (<0.6), the diversity of leaf traits is more likely to drive stability, while under a high aridity level (>0.6), species richness plays a greater role in stability [25]. However, the division of aridity level in this study is quite subjective, and no statistical method is used to identify the aridity threshold. By combining segmented linear regression with moving-window analysis, our study showed that community stability responds nonlinearly to increasing aridity and changes suddenly at an aridity level of 0.88. Our results are similar to those of Berdugo et al. [9], who found that ecosystems may experience an "ecosystem breakdown" phase with an extreme reduction in plant cover when the aridity level >0.83. The transition from grassland ecosystems to desert shrub ecosystems at this critical threshold in China's dryland is of particular concern. In regions with aridity levels >0.88, community stability increased with increasing aridity, possibly due to the extreme sparseness of plants in this region. During the transect investigation, we found that the abundance and richness of species were relatively low, which may lead to small fluctuations in the community in response to disturbances.

Through SEM (Fig. 3a–c), we found that the increase in mean annual precipitation improved community stability by increasing the mean productivity in regions with aridity levels <0.88, which is consistent with previous studies [20,48]. The mean precipitation and precipitation variability drive ecosystem communities' spatial and temporal dynamics [20,21]. A global meta-analysis indicated that increased precipitation enhances the community's temporal stability mainly by increasing the average productivity [48]. In addition, the increase in interannual precipitation variability diminished community stability by increasing the variability of productivity in regions with aridity levels >0.88 (Fig. 3d–f). In these regions, plants are mostly drought-tolerant shrubs with deep roots, enabling them to access deep soil water resources [49] and buffer the effect of mean annual precipitation on the mean productivity. However, increasing variability in interannual precipitation may increase community fluctuations due to lower species diversity and increase variability of community productivity [50], which further affects community stability.

In contrast, the effects of the mean annual temperature and the annual temperature variability on community stability were generally weak in both regions due to water limitation. The increase in variability of annual temperature rather than the mean annual temperature improved community stability in regions with aridity levels <0.88 of China's drylands (Fig. 3a). This result may be because the majority of the sample sites had mean annual temperatures in a limited range of 7–9 °C. The temperature variability amplified the effect of temperature on biomass, which led to an increase in community stability (Fig. 3b). In regions with aridity levels >0.88, neither the mean annual temperature nor the variability in annual temperature had a significant effect on community stability (Fig. 3d), which were different from previous studies. Although some studies indicated that climate warming may reduce community stability [51,52], Liu et al. [52] found that the decrease in community stability is caused by decreased productivity due to increased water limitation by increased temperature. Therefore, the impact of temperature change on community stability likely depends on water constraints. Due to the larger water limitation in regions with the aridity level above 0.88, temperature change may not significantly impact community stability. Overall, in our study area, the impact of temperature on community stability appears to be relatively minor. This could be attributed to the fact that the influence of temperature on the temporal stability of plant communities may depend on water availability, as plant growth is primarily constrained by water availability in drylands.

In regions with aridity levels <0.88, the increase in species richness improved community stability by reducing the variability in productivity (Fig. 3c), while it had non-significant positive effect on the mean productivity in this region (Fig. 3b). In contrast, in regions with aridity levels >0.88, the increase in species richness weakened community stability by increasing the variability in productivity (Fig. 3f), and it had significant positive effect on the mean productivity in this region (Fig. 3e). In regions with aridity levels <0.88, communities with higher species richness had smaller fluctuations in productivity and higher community stability. In response to aridity stress, resource utilization is higher in communities with higher species richness due to ecological niche differentiation promoting species coexistence and facilitating community stability [53]. The results may indicate that complementary effects mainly affect productivity, thereby producing stronger portfolio effects, which reduce productivity variability and further promote community stability in regions with aridity levels <0.88 [14,19]. In contrast, species richness increased the variability in productivity and diminished community stability in regions with aridity levels >0.88. These regions are desert ecosystems dominated by drought-tolerant communities, mostly mono-dominant shrubs. In response to aridity stress, the dominance of high-yield species may increase through the selection effects [14,19]. Based on the SLA data measured, we also found that in this region, the SLA of the dominant species increased with species richness (Fig. S4). Generally, plants with higher SLA have higher photosynthetic capacity, productivity, and lower tolerance [54,55]. Thus, compared to communities with low species richness, the dominant population has higher productivity but relatively lower tolerance in communities with high species richness, which also promotes the variability of community productivity [54]. Moreover, the increase in the dominance of high-yielding species led to a decrease in community evenness, which weakened the portfolio effect and ultimately increased the variability in productivity [12,18]. These results are similar to those of the community model by Wang et al. [18]. Therefore, these results may indicate that selection effects mainly affect productivity, thereby diminishing community stability in regions with aridity levels >0.88.

Soil properties are important abiotic factors affecting plant growth and vegetation distribution, which can further influence community stability. In regions with aridity levels <0.88, a higher soil clay and organic carbon content resulted in greater community stability (Fig. 3a–c). In this region, the water and temperature conditions were relatively suitable for plant growth, which resulted in high plant productivity and carbon input into the soil. Meanwhile, the high clay content facilitated soil organic carbon accumulation [56]. Higher soil organic carbon positively feeds back to plants, increasing species richness and community stability. However, the dominant climatic conditions in this region might mask the effect of soil organic carbon on community stability, leading this effect to be non-significant (Fig. 3a). In regions with aridity levels >0.88, however, communities with higher soil organic carbon contents had greater variability in productivity and lower community stability. This is possible because biomass accumulated in wet years declined more rapidly in dry years for communities with higher soil organic carbon contents (Fig. S5). In addition, occasional precipitation may increase microbial activity due to the priming effect [57,58]. Specifically, microorganisms exhibit high sensitivity to environmental changes, which can induce growth, metabolism, and distribution shifts, enabling them to utilize more organic carbon. Thus, the communities with high soil clay contents have lower soil organic carbon contents, which improves the stability of these communities in regions with aridity levels >0.88 (Fig. 3d–f).

### 4.2 Guidance for ecosystem management

Our study indicated precipitation was the main climatic factor influencing community stability in the drylands of China (Fig. S6). Climate models predict that extreme precipitation in China's drylands will likely increase in the future, and moderate to heavy rainfall and rainstorm events will occur more frequently, especially in the northwest [31,59]. Moreover, the frequency of drought events also may increase in the future [59]. In the face of deteriorating climatic conditions in the future, the community stability of drylands in China might be drastically reduced, according to our results, especially in regions with aridity levels below 0.88. This reduction may have irreversible adverse effects on dryland ecosystems' structure, function, biodiversity, and soil properties [60], leading to land degradation and desertification. Our results indicate that ecosystems with the aridity level around 0.88 are extremely unstable and need to be protected and managed as a priority. Therefore, the adverse impact of future climate change on ecosystem stability should be considered in the ecosystem management of China's drylands.

Facing the intensification of drought and extreme climate events in the future, one of the main purposes of dryland ecological restoration is to establish a relatively stable plant community in terms of community structure and function under deteriorating environmental conditions [61]. A stable plant community has low variability, deviates only slightly from its average state in the case of environmental change, and can return to its equilibrium state quickly after disturbances [62]. Our result suggests that plant diversity is an important biological factor for establishing a stable plant community under climate change, highlighting the need to enhance the protection and restoration of plant communities in the drylands of China. Although implementing the afforestation projects in China has generally greened vast regions of China's dryland, plant diversity has not been considered a key factor in implementing these projects.

**Fig. 4:**
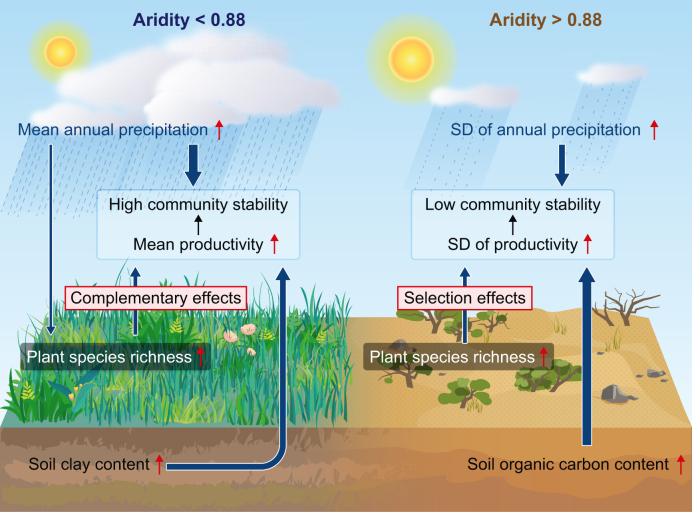
Conceptual diagram of the impact of driving factors on community stability above and below the threshold.

Hence, the ecological restoration project of drylands should be adjusted to improve community stability. Considering the divergent effects of species richness on community stability along the arid gradient, we suggest that in relatively humid regions (i.e., aridity levels <0.88), the richness and evenness of plant communities should be considered, whereas monocultures should be avoided in the process of ecological restoration in drylands of China. Specifically, enclosure or fallowing methods can be employed to restore the plant diversity of pastures or farms to promote ecological niche differentiation, thereby increasing community stability. Simultaneously, using native plants instead of water-intensive fast-growing plants is recommended to prevent soil dryness and facilitate soil moisture recovery. By contrast, in regions with aridity levels >0.88, single species with a high tolerance should be planted first in the early stage of plant community construction or restoration, and more species should be gradually and evenly allocated after the local environment is improved. Specifically, it is advisable to prioritize planting drought-resistant shrubs such as *Haloxylon* spp., *Calligonum* spp., and *Nitraria* spp. to serve as windbreaks and sand stabilizers, gradually improving the local environment. More importantly, the driving mechanisms of community stability under different levels of aridity should be considered for the protection and restoration of drylands in a changing climate (Fig. 4).

### 4.3 Limitations and uncertainties

Vegetation is sparse in drylands, which may increase the uncertainty of satellite observations of vegetation indexes (e.g., the NDVI) [63], especially for those with coarse solutions. Some studies have shown the uncertainties of using NDVI to detect the trend of vegetation growth and change under frequent drought conditions in northwest China [41]. Although it has become common to explore the impact of ecological factors on ecosystem functions by combining observational data with remote sensing data [10,17,25], the analysis of vegetation change using the NDVI needs to be carried out with caution for regions with sparse vegetation due to the interference of the soil background [64]. Furthermore, a recent study showed that above- and below-ground biodiversity drives ecosystem stability in natural alpine grasslands on the Qingzang Plateau [10]. Thus, ignoring the impact of belowground organisms in our study may underestimate the role of biodiversity on ecosystem stability. Some studies have revealed that alterations in ecosystem stability, driven by biodiversity and environmental heterogeneity, are influenced by changes in latitude [65].

Consequently, integrating landscape or topographical factors in future investigations can enhance our comprehension of shifts in ecosystem stability. Despite the above limitations, our research methodology indicates a more robust threshold identification approach that considers the influence of covariates along the aridity gradient on ecosystem stability. Our research results on the ecosystem stability and driving mechanism changes of dryland ecosystems, especially the understanding of different mechanisms in the region above and below a threshold value, can provide targeted and adaptive guidance for ecosystem management and ecological restoration.

## 5 Conclusions

An aridity threshold value leading to abrupt changes in community stability in the drylands of China was detected at an aridity level of 0.88. The impact and underlying mechanisms of ecological and environmental factors on community stability diverge markedly across this threshold. In particular, species richness played an opposite role. In regions with aridity levels below 0.88, the influence of each factor on community stability is primarily mediated through mean productivity. Here, climatic conditions are the most influential, with species richness promoting mean productivity by complementary effects, thereby improving community stability. In contrast, each driver affected community stability mainly by influencing the variability of productivity in regions with aridity levels above 0.88. Soil properties become the critical factor in this context, with species richness increasing the variability of productivity, which in turn leads to reduced community stability. Detecting the threshold and identifying the divergent driving mechanisms in the community stability of dryland ecosystems can help develop adaptive measures to cope with aridification stress and provide guidance for sustainable ecosystem management. Furthermore, the roles of soil organisms in maintaining ecosystem stability through the plant–soil feedback should be incorporated into future research.

## CRediT authorship contribution statement

**Kai Wang:** Conceptualization, Methodology, Formal Analysis, Investigation, Data Curation, Visualization, Writing - Original Draft. **Cong Wang:** Conceptualization, Investigation, Data Curation, Writing - Review & Editing, Supervision. **Bojie Fu:** Writing - Review & Editing, Project Administration, Funding Acquisition. **Jianbei Huang:** Writing - Review & Editing. **Fangli Wei:** Writing - Review & Editing. **Xuejing Leng:** Writing - Review & Editing. **Xiaoming Feng:** Writing - Review & Editing, Project Administration, Funding Acquisition. **Zongshan Li:** Investigation, Writing - Review & Editing. **Wei Jiang:** Investigation, Writing - Review & Editing.

## Declaration of competing interest

The authors declare that they have no known competing financial interests or personal relationships that could have appeared to influence the work reported in this paper.
