# Supplementary material for: Divergent driving mechanisms of community temporal stability in China's drylands
Source: Environ Sci Ecotechnol. 2024 Mar 1;20:100404. doi: 10.1016/j.ese.2024.100404 (PMC10997951; doi:10.1016/j.ese.2024.100404)
Supplement: Multimedia component 1 [file mmc1.docx]

**APPENDIX S1– DATA SOURCES**

Du, Z., He, Y., Fang, P., Duan, Y., & Lu, K. (2020). Community composition, plant species diversity and soil nutrient content of endangered plant *Tetraena mongolica Maxim* (in Chinese)*.* *Chinese Journal of Ecology, 39*(11), 3537-3548. DOI: 10.13292/j.1000-4890.202011.016

Hu, W., Ran, J., Dong, L., Du, Q., Ji, M., Yao, S., . . . Deng, J. (2021). Aridity-driven shift in biodiversity-soil multifunctionality relationships. *Nature Communications, 12*(1), 5350. DOI: 10.1038/s41467-021-25641-0

Jia, Z., Yang, J., Sun, Y., Chen, Q., Yan, R., & Li, N. (2021). Analysis of Species Diversity and Regulation Factors of *Salsola passerina* Community in Alxa Plateau (in Chinese). *Chinese Journal of Grassland, 43*(6), 1-9. DOI: 10.16742/j.zgcdxb.20200419

Liu, W., Li, X., Huang, W., Ma, H., Ma, H., & Wang, W. (2021). Community diversity, patterns of productivity, and factors influencing them in *Stipa* in Ningxia grassland (in Chinese). *Acta Prataculturae Sinica, 30*(1), 12-23.
