# Supplementary material for: Divergent driving mechanisms of community temporal stability in China's drylands
Source: Environ Sci Ecotechnol. 2024 Mar 1;20:100404. doi: 10.1016/j.ese.2024.100404 (PMC10997951; doi:10.1016/j.ese.2024.100404)
Supplement: Multimedia component 2 [file mmc2.docx]

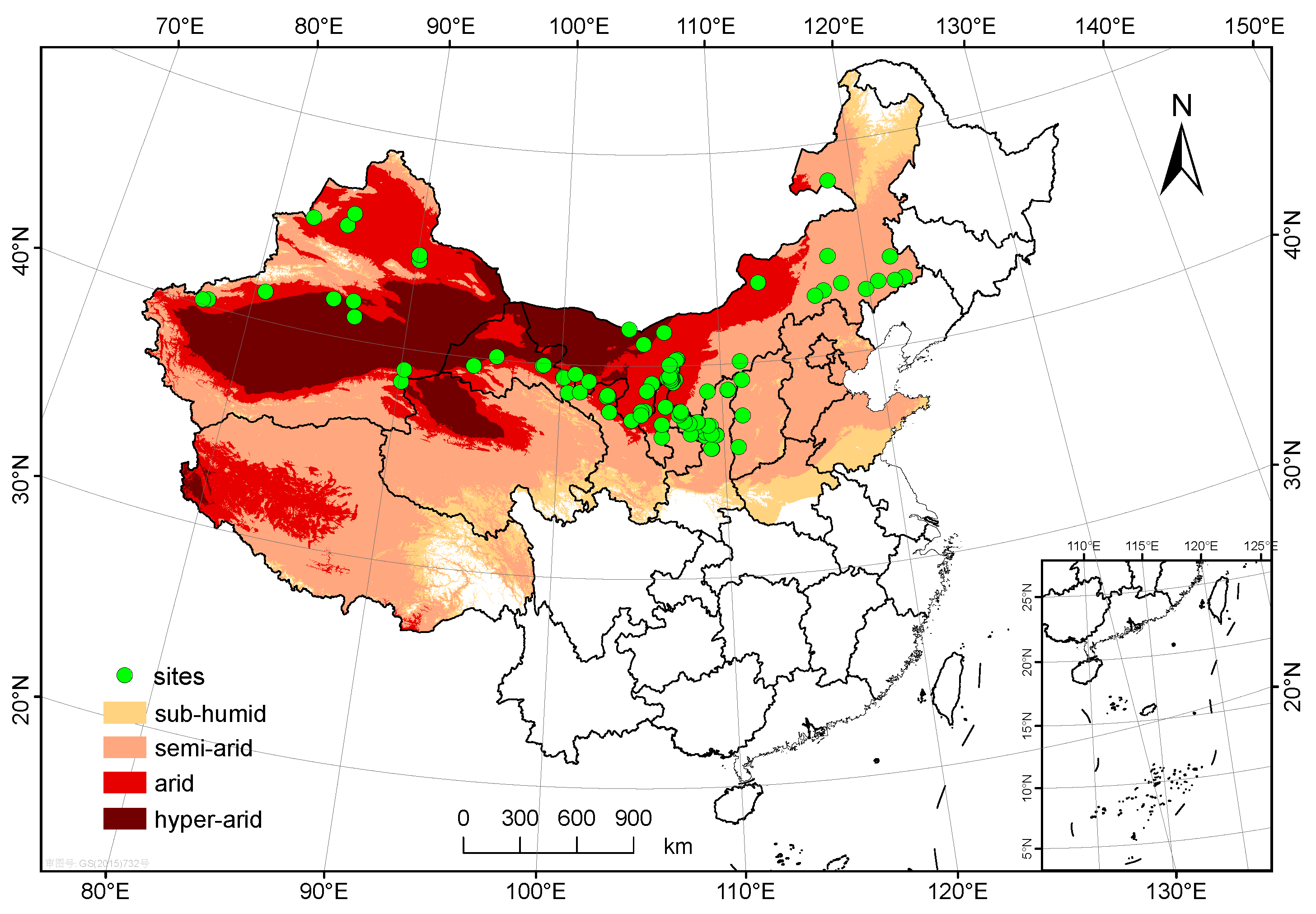


**Fig. S1** Distribution of sampling sites in drylands of China


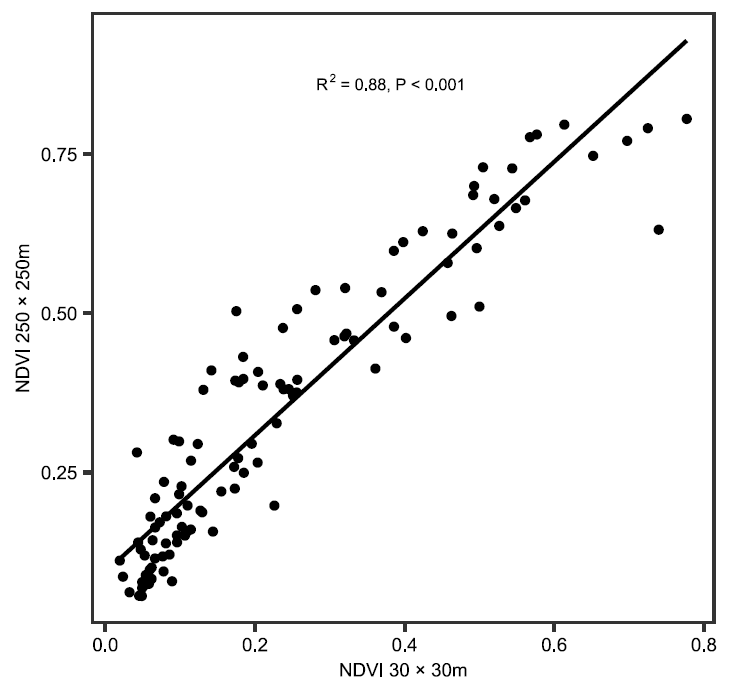


**Fig. S2** Relationship between the NDVI using a pixel size of 30 × 30m and the NDVI using a pixel size of 250 × 250m. The data of 30 × 30m was gathered form the Sentinel-2 Multispectral Instrument and the data of 250 × 250m was gathered from the MOD13Q1 product.


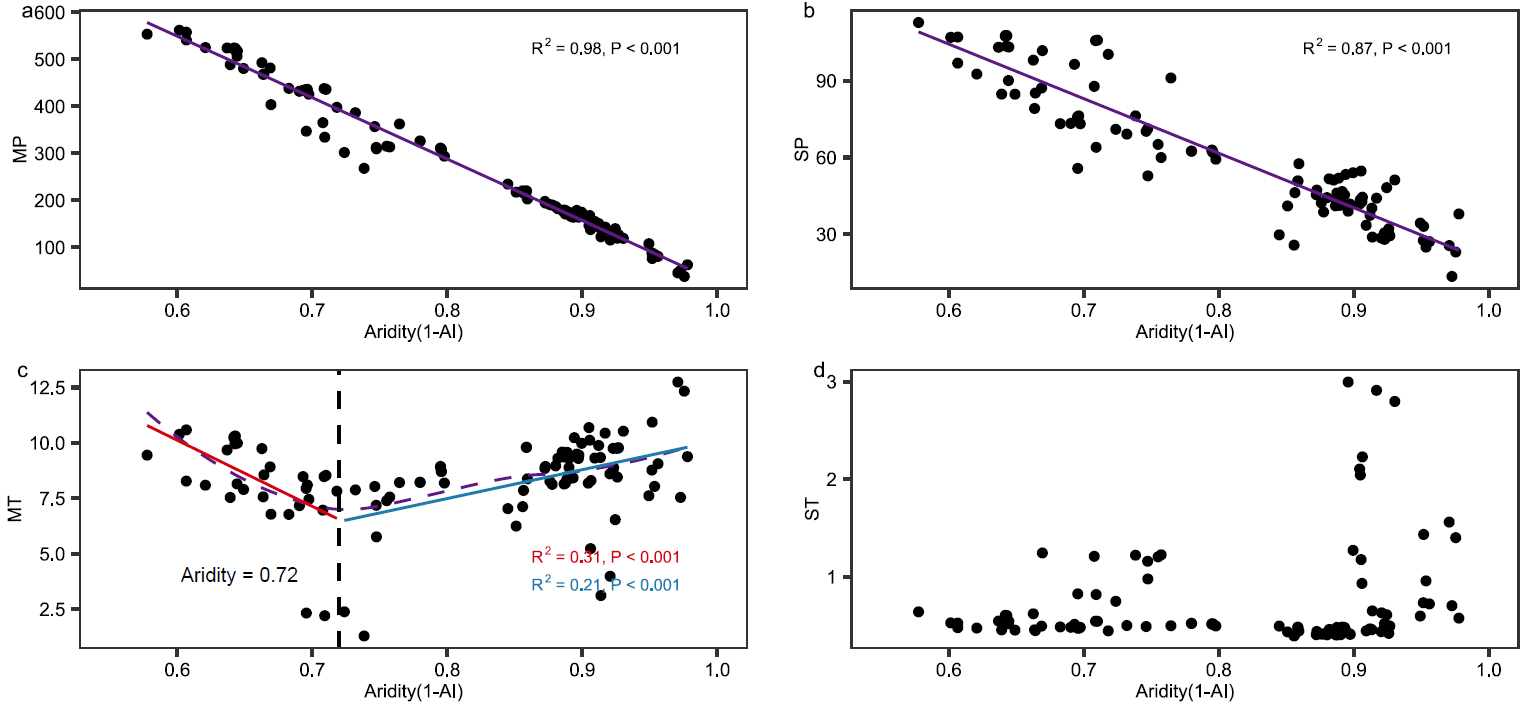


**Fig. S3** The response of climate to increasing aridity. The variation in (a) MP (mean annual precipitation), (b) SP (SD of annual precipitation), (c) MT (mean annual temperature), and (d) ST (SD of annual temperature) with increasing aridity. The purple solid line and the purple dashed line indicate the overall linear regression fitting curves and the overall locally weighted regression fitting curves, respectively. The red and blue solid lines indicate the linear regression fitting curves above and below the threshold, respectively.


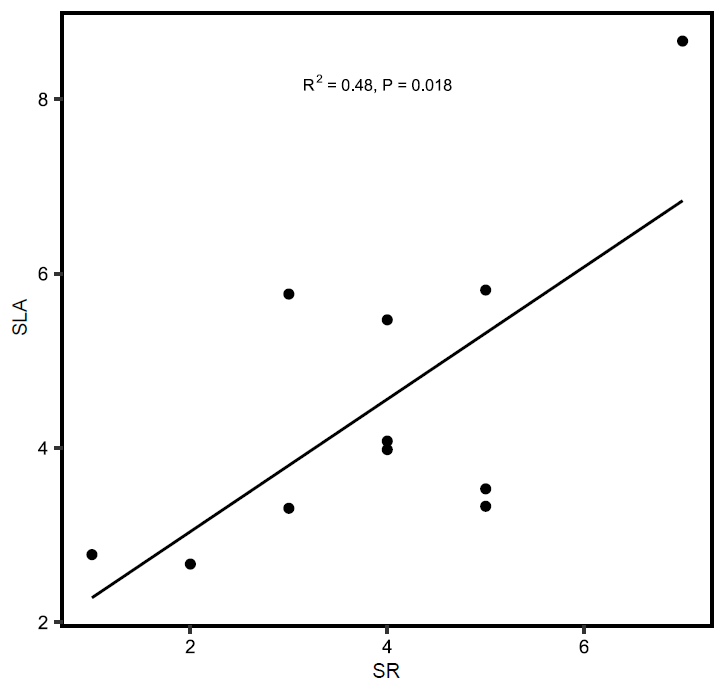


**Fig. S4** Aridity>0.88, linear relationship between species richness and dominant species-specific leaf area. SR = species richness, SLA = specific leaf area.


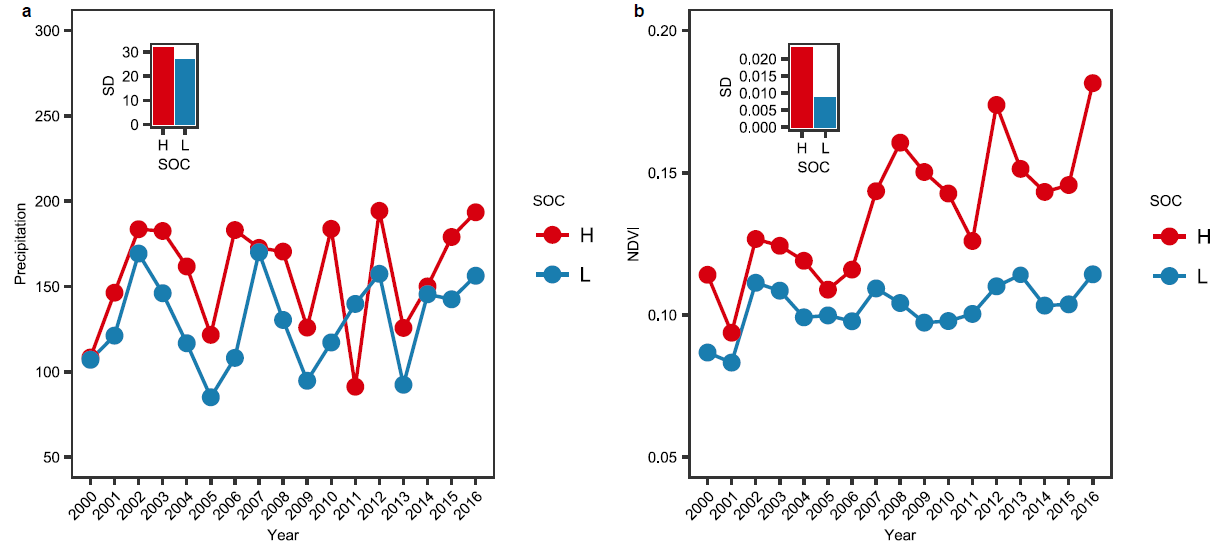


**Fig. S5** Aridity>0.88, differences in precipitation (a) and NDVI (b) with year for communities with different soil organic carbon (SOC). H = communities with high SOC, L = communities with low SOC. The communities with the top 20% of SOC were defined as high SOC communities, and the bottom 20% were defined as low SOC communities. The red bars and dashes represent communities with high SOC, while the blue bars and dashes represent communities with low SOC.


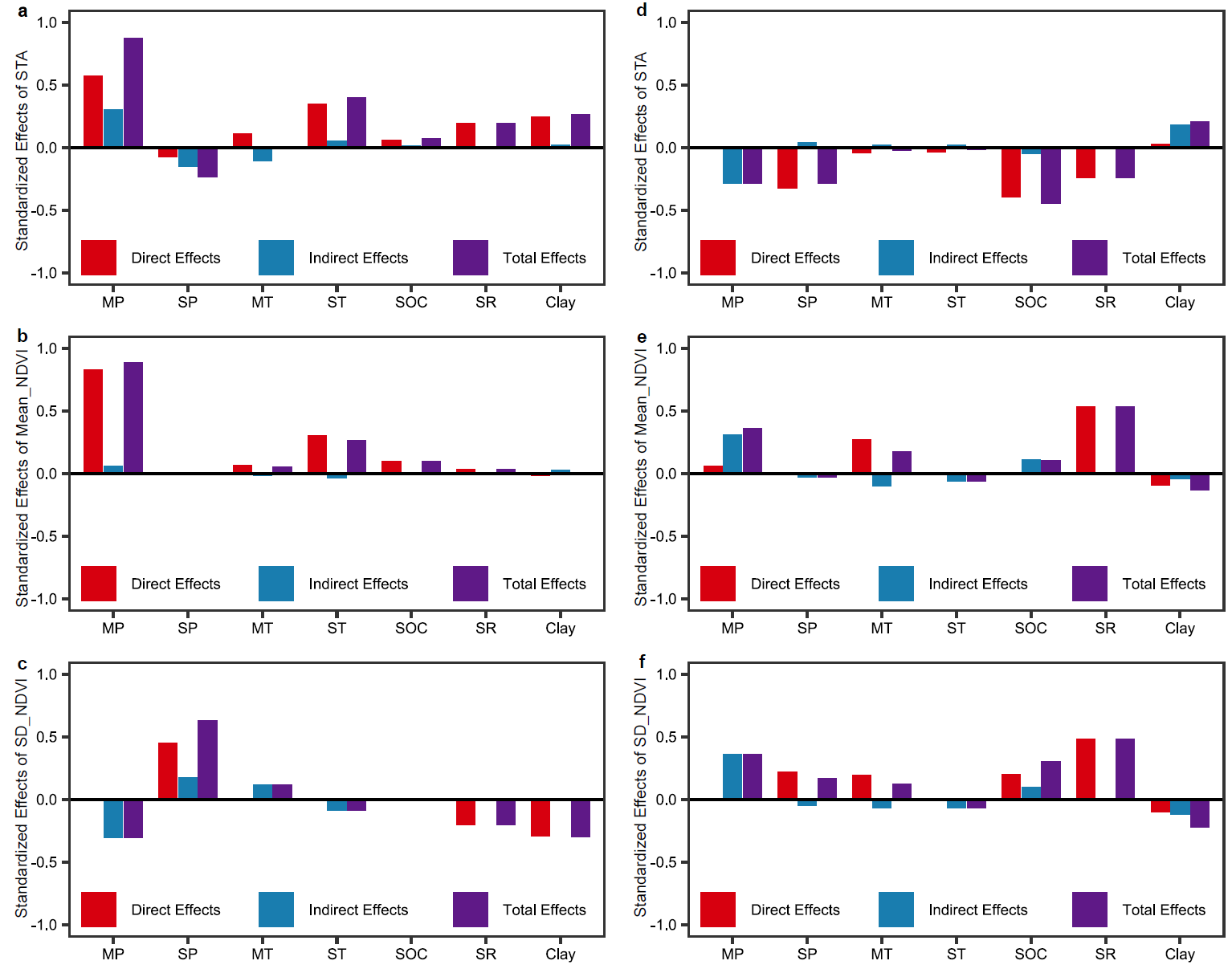


**Fig. S6** Direct effects, indirect effects and total effects of climate, species richness and soil properties on community stability and its components. Aridity<0.88, direct effects, indirect effects and total effects of climate, species richness, and soil properties on (a) community stability (STA), (b) the mean peak NDVI (Mean_NDVI), and (c) the SD of the peak NDVI (SD_NDVI). Aridity>0.88, direct effects, indirect effects and total effects of climate, species richness, and soil properties on (d) community stability (STA), (e) the mean peak NDVI (Mean_NDVI), and (f) the SD of the peak NDVI (SD_NDVI).
